# Prevalence of osteoporosis in the Italian population and main risk factors: results of *BoneTour* Campaign

**DOI:** 10.1186/s12891-016-1248-8

**Published:** 2016-09-17

**Authors:** Loredana Cavalli, Andrea Guazzini, Luisella Cianferotti, Simone Parri, Tiziana Cavalli, Alessia Metozzi, Francesca Giusti, Caterina Fossi, Dennis M. Black, Maria Luisa Brandi

**Affiliations:** 1 Department of Surgery and Translational Medicine, University of Florence, Viale Pieraccini, 6-50139 Florence, Italy; 2 Department of Education and Psychology and Center for the Study of Complex Dynamics, VirtHuLab, University of Florence, Florence, Italy; 3 Department of Epidemiology and Biostatistics, University of California, San Francisco, CA USA

**Keywords:** Osteoporosis, Fracture, Prevention, Epidemiology, Risk factors, FRAX, QUS

## Abstract

**Background:**

*BoneTour* is a campaign conducted throughout the Italian territory for the assessment of Italian people bone status and for the prevention of osteoporosis.

**Methods:**

A total of 7305 sequential subjects of both sexes were screened, collecting clinical data through the FRAX™ questionnaire, and measuring heel bone stiffness by Quantitative Ultrasonography (QUS). The 10-year risk for hip and major osteoporotic fractures was calculated taking into account personal or family history of fragility fracture, smoking, alcohol abuse, rheumatoid arthritis, prolonged steroids assumption. Additional risk factors were evaluated, including early menopause, poor sunlight exposure, low dietary calcium intake, physical inactivity, number of pregnancies, months of lactation, tobacco cigarettes smoked per year, specific causes of secondary osteoporosis. Through a correlation study, the influence of each factor on the development of osteoporosis was analyzed.

**Results:**

As many as 18 % of women suffer from osteoporosis, as defined by QUS T-score. The calculation of FRAX™ confirmed the weight of the already known risk factors. The correlation study revealed the significance of some additional factors, such as hyperthyroidism, nephrolithiasis, Crohn disease, ulcerative colitis, celiac disease, poor sun exposure, and oophorectomy before age 50.

**Conclusions:**

The high prevalence of secondary osteoporosis in the Italian population clearly indicates the importance of additional risk factors not yet included in the FRAX™ algorithm, for which preventive measures should be considered. Screening campaigns may allow both early diagnosis and access to treatment.

## Background

Osteoporosis, estimated to affect 22 millions of women and 5.5 millions of men in the European Union in 2010 [1], is known as *the silent epidemic* because it does not manifest until a fracture occurs. Despite advances in risk assessment and treatment, osteoporosis is still often either not recognized or untreated.

FRAX™ is a widely used risk algorithm, developed by the World Health Organization to predict the 10-year risk of hip and major osteoporotic fractures [2–4]. FRAX includes a concise set of clinical risk factors (e.g., Age, sex, BMD, etc.) but dietary factors and other chronic diseases potentially affecting bone mass and quality are not included. In case DXA BMD is not available, FRAX can be calculated based on non- BMD risk factors only.

In many parts of the world, in some countries and in some less well-served areas, DXA is not easily available and therefore some alternative measurements of bone strength, i.e., ultrasound, have been suggested as a practical alternative to help raise awareness of the disease [5–9].

In this report, the results of a large epidemiological campaign conducted via a mobile van carried out throughout Italy in 2011 and 2012, called “*BoneTour”,* are described.

There are 3 principal aims of BoneTour campaign using a motor home: i) to carry out a population screening in order to identify individuals at risk for osteoporosis and to educate them; ii) to obtain National epidemiological results that could enable the development of prevention measures aimed at groups at risk and iii) to correlate individual risk factors with bone quality, assessed by Quantitative Ultrasonography (QUS), as well as previous fragility fractures.

## Methods

### BoneTour campaign and recruitment of participants

Data deriving from a screening campaign promoted by Fondazione Italiana Ricerca Malattie Ossee (F.I.R.M.O.), a non-profit organization devoted to the study of bone diseases, is reported in this paper. The study was approved by the Internal Review Board of the University of Florence. Subjects of both sexes were recruited on a voluntary basis in major Italian cities as representative of the different regions of belonging. Thus, no age restrictions or recruitment criteria were applied.

The evaluation was carried out on May-June 2011 and on September 2012 in a mobile ambulatory (motor home) with a staff composed by physicians, technicians and nutritionists. Subjects were administered the “One Minute Risk Test” questionnaire (https://www.iofbonehealth.org/sites/default/files/PDFs/2012-IOF_risk_test-english[WEB].pdf), including FRAX™ risk factors and additional questions on different causes of secondary osteoporosis (i.e., hyperthyroidism, calcium nephrolithiasis, celiac disease, Crohn’s disease, and ulcerative colitis). Further information regarding number of pregnancies, months of lactation, age of menopause, and quantified cigarette smoking (pack-years) were also collected.

Once the individual risk of fracture was established, the medical staff handed out practical advice for the prevention of osteoporosis. After the obtainment of an informed consent, data were anonymously reported into an electronic database to allow pooled analysis.

### Assessments and measurements

Quantitative ultrasonometry (QUS) was performed by Achilles Insight (Lunar, GE), a portable, easy-to-operate, radiation-free device suitable for low-cost, rapid, large screening of bone status (bone density, structure and composition) [10, 11]. Since many studies demonstrated the association of QUS results with BMD measured by DXA, both in men and post-menopausal women, as well as QUS effectiveness in identifying osteoporotic subjects and estimating their risk of femoral fracture, subjects have been classified as normal, osteopenic and osteoporotic according to ultrasound T-score (> −1, between −1 and −2.4, and ≤ −2.5, respectively) [12–16].

**Fig. 1:**
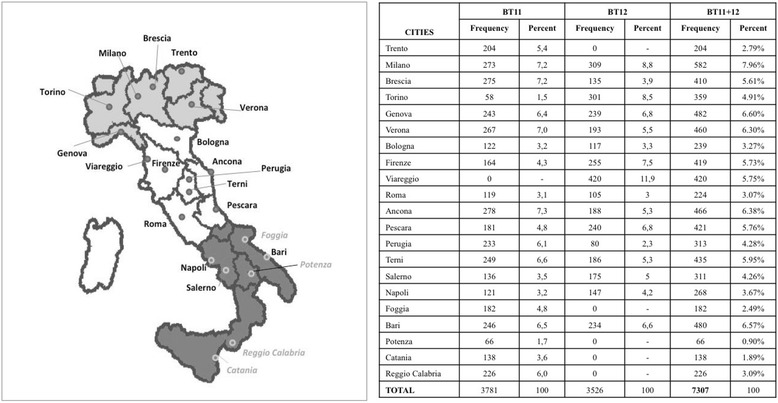
Geographic distribution of 2011 and 2012 BoneTour campaigns (BT11 and BT12, respectively), with frequency and percentual distribution

The 2011 BoneTour (BT11) and the 2012 BoneTour (BT12) campaigns were carried out in 3779 individuals (belonging to 20 cities) and in 3526 subjects (17 cities) respectively (Fig. 1). In BT12, weight and height were also measured by a mechanical scale with an altimeter aboard the motor home and used to calculate the body mass index (BMI).

The questionnaire investigated the risk factors for which numerous evidence support premenopausal and peri-menopausal women screening: prolonged use of corticosteroids (≥5 mg of prednisone for 3 months or longer), protracted amenorrhea, rheumatoid arthritis (RA), and prolonged hyperthyroidism [17, 18]. As regards hyperparathyroidism (HPT), this being a condition often unrecognized by the patient, we investigated the history of recurrent and/or familiar nephrolithiasis, which can be associated with loss of BMD even when not due to HPT [19].

In the assessment of *modifiable risk factors*, some data were provided through a dichotomous question (presence of diseases affecting bone mass, history of oophorectomy before age 50). Other quantitative data were collected in the form of continuous variable, such as BMI, age of menopause, estimated average number of cigarettes smoked per year (reported as “pack-years”: the average number of daily cigarette multiplied by the number of years of smoking). Further data derived from a question detecting the overcoming of a threshold for a healthy behavior: “Is your daily level of physical activity less than 30 min per day?”; “Do you regularly drink more than 2 units of alcohol a day?”; “Do you spend less than 10 min per day exposed to sunlight?”

### Data analysis

The results of the survey were pooled for the epidemiological macroarea analysis, since the local samples were homogeneous in size and characteristics. These data were afterwards examined in order to calculate the 10-years risk for hip (FRAX-B) and major osteoporotic fractures (FRAX-A), by considering the abovementioned specific risk factors associated with weight and height values [20]. FRAX-A and FRAX-B were calculated for each subject by entering in the algorithm FRAX® version 3.7 Italy model the following data: age, sex, weight (kg), height (cm), previous fracture, parent hip fracture, smoking, glucocorticoids, RA, secondary osteoporosis, and alcohol intake ≥3 units/day. Results are expressed as the mean ± standard deviation.

The continuous FRAX™ indicators were discretized: the cases characterized by a FRAX™ value greater than the average were labeled with the value “1”, the others with “0”. Exploratory analysis of the database was performed with the Statistical Package for Social Sciences (SPSS) software release 20.0. Given the difference of sex-frequency distribution, the comparisons operated on the base of sex and history of fragility fracture were preprocessed using a bootstrap sampling in order to balance the subsets. The Skewness and the Kurtosis of the distribution allowed the use of parametric tests as inferential statistics, the process of drawing conclusions from a dataset, which is subject to random variation. Finally, the analysis of Pearson correlations was carried out between continuous risk factors and clinical quantitative parameters assumed as describing bone health status (FRAX-A, FRAX-B, Z-score and T-Score). As a general procedure, the continuous observables have been discretized properly according to the adopted statistical analysis, if the technical requirements were fulfilled. In order to satisfy the conditions required from the parametric statistics taking into account the dimension of the study, a Monte Carlo method of Bootstrap was applied for each variable in order to extract subsamples of comparable size, followed by the execution of the *t test* or of the ANOVA test.

## Results

### Study group

The demographic features of the experimental sample (BT11 and BT12, total range *n* =7305 subjects) are presented in Tables 1 and 2 with respect of their gender- and age-related frequency distribution.

**Table 1:** Description of the screened population: prevalence of nominal risk factors in the general screened population and by sex (as expressed in % and absolute number *n* of the total subjects, of females and of males respectively) scalar parameters (mean ± SD)

	All	Females	Males
Sex	100 % *n* =7305	83.7 % *n* =6114	16.3 % *n* =1191
Family history of osteoporosis	25.2 % *n* =1839	26.2 % *n* =1605	19.6 % *n* =234
Previous fracture	18.3 % *n* =1335	18.2 % *n* =1115	18.4 % *n* =220
Underweight	8.6 % *n* =626	8.7 % *n* =535	7.6 % *n* =91
Steroids	11.6 % *n* =849	11.8 % *n* =725	10.4 % *n* =124
Rheumatoid Arthritis	6.6 % *n* =485	6.8 % *n* =416	5.7 % *n* =69
Nephrolithiasis^a^	5.5 % *n* =193	5.8 % *n* =175	3.5 % *n* =18
Hyperthyroidism^a^	2.8 % *n* =100	2.9 % *n* =89	2.15 % *n* =11
Early menopause^b^	-	23.9 % 885	-
Amenorrhea > 12 months	-	7.3 % 446	-
Ovariectomy by age 50	-	6.8 % 418	-
Alcohol consumption	4.4 % *n* =323	3.1 % 191	11.1 % 132
Smoking habit	37.9 % *n* =2772	35.1 % 2151	52.1 % 621
Exercise < 30 min/day	19.0 % *n* =1390	19.1 % 1169	18.5 % 221
Avoiding dairy products	15.8 % *n* =1157	25.6 % 979	15.0 % 178
Exposure to sunlight	24.3 % *n* =1777	25.6 % 1564	17.9 % 213
Crohn disease^a^	0.2 % *n* =6	0.2 % *n* =5	0.2 % *n* =1
Celiac disease^a^	0.6 % *n* =22	0.7 % *n* =20	0.4 % *n* =2
Rectocolitis ulcerosa^a^	0.3 % *n* =12	0.3 % *n* =10	0.4 % *n* =2
Secondary osteoporosis^a^	3.9 % *n* =138	4.1 % *n* =124	2.7 % *n* =14
Age (years)	58.3 ± 12.4	57.6 ± 12.0	62.00 ± 13.6
BMI^a^	24.6 ± 4.01	24.42 ± 4.2	25.86 ± 3.5
Menopause age (years)^b^	-	49.41 ± 8.4	-
Pregnancies (number)	-	1.29 ± 1.1	-
Lactation (months)	-	8.63 ± 44.8	-
HRT (months)^b^	-	5.75 ± 20.2	-
Smoking (pack/years)	5.9 ± 11.07	5.26 ± 10.4	10.17 ± 16.8
Mean QUS T-score	−0.98 ± 4.04	−1.13 ± 4.4	−0.23 ± 4.6
Mean QUS Z-score	0.17 ± 2.01	0.07 ± 1.9	0.67 ± 3.2
Mean FRAX-A score^a^ (% value)	7.87 ± 7.09	8.22 ± 8.3	5.79 ± 4.6
Mean FRAX-B^a^ score (% value)	3.04 ± 5.09	3.14 ± 6.2	2.43 ± 3.7
Prevalence of QUS T-score < −2.5	18,1 % 1212	20,2 % 1128	7,4 % 84
Prevalence of QUS Z-score < −2.5	1,1 % 76	1 % 58	1,6 % 18
Prevalence of FRAX-A score <10 %	77 % 2710	75,4 % 2266	87,1 % 444
Prevalence of FRAX-B score <3 %	75,8 % 2653	75,7 % 2264	76,3 % 389

^a^As calculated in BT12 group (subset of *n* =3526 subjects); ^b^as calculated in the group of postmenopausal women (*n* =3691)

**Table 2:** Distribution of nominal risk factors for the considered age ranges the general screened population and by sex (as expressed in % of the age group and absolute number, *n*, of subjects)

Age ranges (years)	<40	40–49	50–59	60–69	70–79	≥80	Total
%	7.4 %	14.9 %	30.1 %	28.0 %	16.3 %	3.4 %	100 %
Count	(*n* =541)	(*n* =1087)	(*n* =2198)	(*n* =2043)	(*n* =1191)	(*n* =245)	(*n* =7305)
%	84.5 %	87.3 %	90.1 %	82.4 %	74.1 %	65.3 %	83.7 %
Female sex	(*n* =457)	(*n* =949)	(*n* =1981)	(*n* =1684)	(*n* =883)	(*n* =160)	(*n* =6114)
%	29.7 %	30.5 %	26.5 %	23.2 %	21.3 %	13.9 %	25.17 %
Family history	(*n* =161)	(*n* =332)	(*n* =584)	(*n* =475)	(*n* =253)	(*n* =34)	(*n* =1839)
%	12.0 %	13.6 %	16.3 %	18.6 %	24.6 %	36.3 %	18.3 %
Previous fracture	(*n* =65)	(*n* =148)	(*n* =358)	(*n* =381)	(*n* =293)	(*n* =89)	(*n* =1334)
%	15.3 %	9.8 %	8.2 %	6.0 %	8.9 %	10.6 %	8.5 %
Underweight	(*n* =83)	(*n* =107)	(*n* =180)	(*n* =123)	(*n* =106)	(*n* =26)	(*n* =625)
%	10.5 %	12.5 %	10.7 %	11.1 %	13.3 %	13.5 %	11.6 %
Steroids	(*n* =57)	(*n* =136)	(*n* =236)	(*n* =228)	(*n* =158)	(*n* =33)	(*n* =848)
%	2.4 %	5.2 %	5.7 %	7.3 %	9.9 %	9.0 %	6.6 %
Rheumatoid Arthritis	(*n* =13)	(*n* =57)	(*n* =125)	(*n* =150)	(*n* =118)	(*n* =22)	(*n* =485)
%	0.4 %	0.4 %	0.7 %	0.7 %	1.4 %	0.9 %	0.8 %
Nephrolithiasis^a^	(*n* =1)	(*n* =2)	(*n* =8)	(*n* =7)	(*n* =8)	(*n* =1)	(*n* =27)
%	0 %	1 %	1.5 %	1.6 %	2.1 %	0.9 %	1.4 %
Hyperthyroidism^a^	(*n* =0)	(*n* =5)	(*n* =16)	(*n* =16)	(*n* =12)	(*n* =1)	(*n* =50)
%	0.4 %	11.8 %	13.0 %	17.6 %	20.9 %	23.1 %	14.5 %
Early menopause	(*n* =2)	(*n* =112)	(*n* =259)	(*n* =296)	(*n* =185)	(*n* =37)	(*n* =891)
%	8.3 %	8.3 %	8.8 %	5.9 %	5.9 %	4.3 %	7.4 %
Amenorrhea	(*n* =38)	(*n* =79)	(*n* =174)	(*n* =100)	(*n* =52)	(*n* =7)	(*n* =450)
%	0.6 %	3.3 %	5.3 %	9.0 %	12.2 %	11.2 %	6.8 %
Ovariectomy	(*n* =3)	(*n* =31)	(*n* =106)	(*n* =153)	(*n* =108)	(*n* =18)	(*n* =419)
%	5.4 %	3.0 %	4.0 %	4.9 %	5.4 %	2.8 %	4.4 %
Alcohol > 3/die	(*n* =29)	(*n* =33)	(*n* =89)	(*n* =100)	(*n* =65)	(*n* =7)	(*n* =323)
%	38.0 %	35.7 %	42.0 %	38.8 %	32.4 %	30.2 %	37.9 %
Smoking	(*n* =206)	(*n* =388)	(*n* =924)	(*n* =792)	(*n* =386)	(*n* =74)	(*n* =2770)
%	19.6 %	21.4 %	19.0 %	18.5 %	16.6 %	22.0 %	19.0 %
Exercise < 30’/day	(*n* =106)	(*n* =233)	(*n* =419)	(*n* =379)	(*n* =198)	(*n* =54)	(*n* =1389)
%	20.7 %	16.3 %	14.5 %	16.7 %	14.0 %	15.1 %	15.8 %
Avoiding dairy products	(*n* =112)	(*n* =178)	(*n* =320)	(*n* =343)	(*n* =167)	(*n* =37)	(*n* =1157)
%	24.0 %	22.8 %	24.5 %	24.8 %	24.1 %	27.3 %	24.3 %
Sunlight exposure < 10’/day	(*n* =130)	(*n* =248)	(*n* =538)	(*n* =506)	(*n* =288)	(*n* =67)	(*n* =1777)
%	0.0 %	0.2 %	0.3 %	0.1 %	0.2 %	0.0 %	0.2 %
Crohn disease^a^	(*n* =0)	(*n* =1)	(*n* =3)	(*n* =1)	(*n* =1)	(*n* =0)	(*n* =6)
%	0.4 %	0.4 %	0.1 %	0.3 %	0.7 %	0.9 %	0.3 %
Rectocolitis ulcerosa^a^	(*n* =1)	(*n* =2)	(*n* =1)	(*n* =3)	(*n* =4)	(n =1)	(*n* =12)
%	3 %	1.6 %	0.3 %	0.1 %	0.2 %	0.9 %	0.6 %
Celiac disease^a^	(*n* =8)	(*n* =8)	(*n* =3)	(*n* =1)	(*n* =1)	(*n* =1)	(*n* =22)
%	3.4 %	3.7 %	3.6 %	3.5 %	5.7 %	3.7 %	3.9 %
Secondary osteoporosis^a^	(*n* =9)	(*n* =18)	(*n* =39)	(*n* =36)	(*n* =32)	(*n* =4)	(*n* =138)

^a^As calculated in BT12 group (subset of *n* =3526 subjects)

Females largely outnumbered males in the whole sample (*n* =6114, 83,7 %, versus *n* =1191, 16,3 %).

Regarding age, the sample showed a Gaussian distribution. Mean subject age was 58.3 ± 12.4 years (range 17–97), in particular 57.6 ± 12.0 for women (range 17–97) and 62.0 ± 13.6 for men (range 22–92). In the female group, 3691 patients (60.4 %) were postmenopausal.

The percentage of women and men was comparable in the two groups (women: 82.0 % in BT11 vs 85.5 % in BT12; men: 18.0 % in BT11 vs 14.5 % in BT12).

Herein, the analysis of the whole group will be described.

### Bone status according to calcaneus ultrasonography

Regarding BMD, 18.1 % of the study group resulted osteoporotic (age 65.3 ± 11.0, range 22–97), 42.1 % osteopenic (age 59.4 ± 11.3, range 19–89), while 39.8 % normal (age 54.1 ± 12.6, range17-92).

In the women subgroup, 20.2 % were osteoporotic (age 65.2 ± 110.7, range 22–97), 44.1 % osteopenic (age 58.7 ± 11.0, range 19–89), and 35.7 % normal (age 52.0 ± 11.5, range 17–85).

In the men subgroup 7.4 % were osteoporotic (age 67.4 ± 14.1, range 25–92), 32.4 % osteopenic (age 63.9 ± 12.7, range 25–89), and 60.2 % normal (age 60.6 ± 13.5, range 22–92).

### Prevalence of risk factors for osteoporosis

With regard to *non-modifiable risk factors*, in addition to sex and age, genetic predisposition to develop osteoporosis was investigated through the detection of a past or family history of osteoporotic fractures. When asked if one of their parents had shown signs of osteoporosis before the age of 75, such as kyphosis, an important decrease in height or a major fracture occurred in absence of an efficient trauma (fragility fracture), as much as 25.4 % of the interviewees answered “yes”. As much as 18.3 % of the sample reported a previous fragility fracture.

The mean age of menopause was 49.3 ± 10.1 years. Among the group of postmenopausal women (*n* =3691), 23.9 % had premature ovarian failure (i.e., age of menopause ≤ 45 years and negative history for hormonal replacement therapy).

As regards physical exercise, 19 % of the subjects performed inadequate physical activity (i.e., less than 30 min a day).

As much as 38 % of the sample reported current or prior tobacco smoking habit, in particular men. The prevalence of this risk factor was also assessed for subgroups, with a statistically significant difference according to sex (men 52.1 % women 35.1 %, *χ*^2^ = 121.9, *p < 0.01*).

Regarding alcohol intake, sun exposure and dairy products assumption, 4.4 % of all the interviewees consumed more than 2 units a day of alcoholic drinks, 24.3 % expose to sunlight for less than ten minutes a day, 15.8 % avoided dairy products without even assuming any calcium supplement.

Recurrent nephrolithiasis, hyperthyroidism, celiac disease, ulcerative colitis and Crohn’s disease were assessed both singularly and pooled together under the name of “secondary osteoporosis”. As a whole, secondary osteoporosis causes were reported by 3.9 % of the sample (2.7 % men and 4.1 % women).

We also took into account the number of behavioral risk factors concurrent in the same subject, finding that, on average, each person has 2.6 ± 1.2 total risk factors (habits), with an average for the modifiable factors of 1.5 ± 1.0. Moreover, the distribution appeared as not “*Normal*” (i.e., skewedness and kurtosis are not within the range [1, −1]); in particular, a negative exponential-like trend was observed, making the concurrence of factors in the same person less probable when the number of factors increased*.* Thus, the occurrence of multiple risk factors in the same person was a rare event. Only the 0.2 % of the sample examined showed no risk factors at all, while the 31.5 % of the sample had no modifiable risk factors.

FRAX was calculated in the BT12 group, comprising 3526 people, in which body weight and height were known. Mean BMI value resulted 24.6 ± 4.01. Eighty-one out of 3526 subjects had a BMI compatible with underweight, a known risk factor for osteoporosis. Indeed, the 78.6 % of the underweight subjects showed reduced (i.e., T-score < −1) bone stiffness independently of other risk factors; in particular, 30 % of them showed a QUS T-score under −2.5, 48.6 % subjects had osteopenia, and only 21.4 % had a normal T-score.

The average value reported with respect to the FRAX-A was 7.87 ± 7.9, while the FRAX-B resulted of 3.04 ± 5.9. In the female group FRAX-A and FRAX-B were higher than males (FRAX-A: 8.22 ± 8.3 and 5.79 ± 4.6, in women and men, respectively; FRAX-B: 3.14 ± 6.2 and 2.43 ± 3.7 in women and men, respectively). Both the distributions showed a right long tail, indicating how the majority of the sample obtained a score near zero. Thus, 33 % of the BT12 sample (24.6 % of women and 12.9 % of men) reported FRAX-A >10 %, while 24.2 % of BT12 (24.3 % of women and 23.7 % of men) had a FRAX-B >3 %.

### Analysis of the results according to gender, previous fracture and age

A gender analysis was performed for risk factors.

The prevalence of smoking habit resulted significantly higher among men (53.2 %) than women (39.1 %) (Chi^2^ = 35.819, *p* < 0.01). Similarly, the prevalence of declared excessive alcohol consumption appeared strongly different between males (12.3 %) and females (3.1 %) (Chi^2^ = 88.303, *p* < 0.01).

With respect to low sun exposure (i.e., spending less than 10 min outdoors), women resulted significantly less exposed to sunlight than men (26.7 and 19.2 %, respectively, Chi^2^ = 13.220, *p* < 0.01).

The prevalence of risk factors was then assessed with respect to the history of fragility fractures.

The percentage of patients with previous fractures was greater (26.1 %) in subjects with a positive family history, than in people with negative family history of osteoporosis (17.3 %) (Chi^2^ = 22.584, *p* < 0.01).

As much as 16 % of fractured subjects, versus 9.3 % of the non-fractured ones had reported to have taken glucocorticoids for at least 3 consecutive months (Chi^2^ = 24.835, *p* < 0.01).

The positive finding of previous fragility fractures was significantly higher in subjects affected by reumathoid arthritis (4 %) than in unaffected people (2.1 %) (Chi^2^ = 6.941, *p* < 0.01).

With respect to alcohol use/abuse the probability of observing a previous fracture in subjects assuming at least 3 daily units of alcohol was higher (6.7 %) than in those who drink fewer (4 %) (Chi^2^ = 7.335, *p* < 0.01).

The incidence of fractures in women who underwent oophorectomy before age 50 was significantly higher (8.2 %) compared to the sample without this clinical event (5.2 %) (Chi^2^ = 6.246, *p* < 0.01).

The conditions resulting not statistically related to age were: steroid use, hyperthyroidism, calcium nephrolithiasis, physical inactivity, scarce sunlight exposure, ulcerative colitis and Crohn’s disease.

**Fig. 2:**
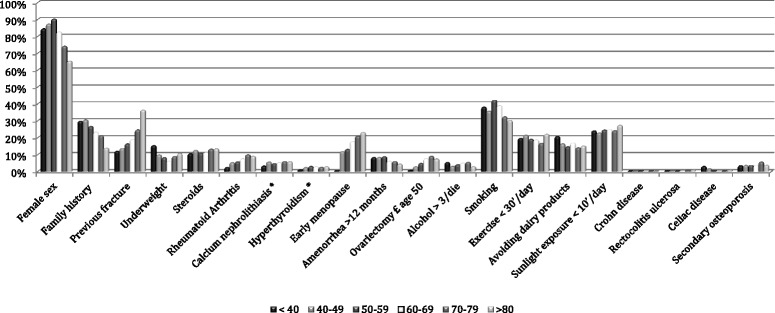
Distribution of nominal risk factors for the considered age ranges expressed in years the general screened population (as expressed in % of the relative age-range for each different considered factor). *As calculated in BT12 group (subset of *n* = 3526 subjects)

In order to analyze the age effect on the observed variables of interest, the age of the subjects was discretized along a six-levels scale (<40 years; 40–49 years; 50–59 years; 60–69 years; 70–79 years; ≥80 years) and the prevalence of osteoporosis risk factors, an analysis of their frequency distribution in the different age ranges was performed (Table 3 and Fig. 2). Conversely, the other known risk factors have a significantly different prevalence according to the age group. Female gender shows the highest proportion in the sixth decade (*χ*^2^ = 222.759, *p* < 0.01). Family history of osteoporosis was mostly reported in the age range 50–59 years (*χ*^2^ = 51.685, *p* < 0.01). A previous fracture was mostly present in the age range 60–69 years, as well as rheumatoid arthritis, scarce dairy products assumption and alcohol abuse, whereas smoking habit is more common in the age group 50–59, like underweight (*χ*^2^ = 52.319, *p* < 0.01); as much as 4.8 % of fractured people in the whole sample is younger than 40.

**Table 3:** Correlations between scalar parameters and discretized age

Risk factor	Discretized age	
	Pearson correlation	Significance
Age at menopause	0.050	*p.* < 0.01
Pregnancies	0.170	*p.* < 0.01
Lactation (months)	0.014	ns
T-score	- 0.134	*p.* < 0.01
Z-score	0.019	*ns*
HRT (months)	0.099	*p.* < 0.01
FRAX-A	0.605	*p.* < 0.01
FRAX-B	0.532	*p.* < 0.01
BMI	0.109	*p.* < 0.01
Smoking pack years	0.064	*p.* < 0.01

In order to estimate the statistical relationship between the gender and the FRAX-A score, given the unbalanced sampling of gender, a discretization procedure should be administered to the continuous variable FRAX-A in the BT12 group. The statistical relations between discretized age and the other risk factors are reported in Table 3.

In particular, when the whole cohort FRAX-A average score (7.87 ± 7.9) has been taken as cut-off/threshold in order to separate the sample into two subgroups, the 10-years risk of major fractures is lower for men (19.4 % = FRAX-A more than average value) than for women (32.9 %) (Chi^2^ = 37.861, *p* < 0.01). Conversely, the average scores FRAX-B does not show any significant difference with respect to gender.

Increasing age results positively correlated to a longer hormone replacing therapy, a major FRAX –A and –B score, a higher BMI, number of pregnancies, and smoking, while it is negatively related to T-score as measured by heel QUS, as expected.

### Parametric inferential statistics

As regards subjects assuming excessive alcohol, they resulted heavier (t = −2.108, *p* < 0.05) and more likely smoker than the non-drinking people (t = −3.042, *p* < 0.01). Noteworthy, they also showed higher T-score values (t = −2.128, *p* < 0.05).

Avoiding dairy products appeared associated with a greater number of packs of cigarettes smoked per year (t = −2.159, *p* < 0.05). Women who did not take dairy products reported a longer duration of hormone replacement therapy after menopause (t = −2.045, *p* < 0.05).

As expected, people affected by celiac disease were younger (t = 2.271, *p* < 0.05), lighter (t = 2.561, *p* < 0.05) and with a lower BMI compared to people without celiac disease (t = 2.844, *p* < 0.01).

Considering physical exercise, people who declared to do at least 30 min of daily activity was significantly leaner (t = −3.788, *p* < 0.01) and with a lower pack-years value (t = −2.714, *p* < 0.01) than sedentary people.

Subjects who exposed themselves to sunlight for less than 10 min per day showed an older age (t = −2.152, *p* < 0.05), a higher BMI (t = −2.201, *p* < 0.01), and more importantly a more elevated FRAX-A (t = −2.210, *p* < 0.05) and –B risk (t = −1.925, *p* = 0.05).

Women reporting history of amenorrhea protracted more than twelve consecutive months resulted younger than those without this risk factor (t = 3.045, *p* < 0.01).

The cases of nephrolithiasis showed a significantly lower T-score (t = 2.041, *p* < 0.05) and Z-score (t = 2.351, *p* < 0.05), as well as a higher FRAX-A (t = −2.028, *p* < 0.05).

People who had suffered from hyperthyroidism had a higher FRAX-A (t = −3.3, *p* < 0.01) and FRAX–B values (t = −2.817, *p* < 0.01), and were older than people without this endocrine disorder (t = −2.544, *p* < 0.05).

People with a previous fracture resulted heavier (t = −3.692, *p* < 0.01) and older (t = −5.171, *p* < 0.01) than the non-fractured people, and showed a lower T-score (t = 3.089, *p* < 0.05) and a higher number of concurrent risk factors (t = −2.083, *p* < 0.05).

The presence of secondary osteoporosis, encompassing a positive medical history of Crohn’s disease, ulcerative colitis, celiac disease, hypercalciuria or hyperthyroidism, was associated with lower T- (t = 3.073, *p* < 0.01) and Z-scores (t = 2.696, *p* < 0.01), as well as with higher FRAX-A (t = −4.684, *p* < 0.01) and FRAX–B values (t = −4.119, *p* < 0.01).

Women who underwent ovariectomy by age 50 resulted to have a higher FRAX-A risk (t = −2.276, *p* < 0.05). Noteworthy the duration of lactation did not show any significant correlation with osteoporosis.

Through a multinomial logistic regression, each risk factor was analyzed related to discretized T-score (classified as lower or higher than mean T-score value, i.e., −0.98) and results are reported in Table 4. Among factors belonging to FRAX algorithm, female gender, family history of osteoporosis, previous fracture, low body weight, use of steroids, smoking habit, secondary osteoporosis resulted significantly associated with a lower T-score; among the other risk factors, the presence of one of the following factors including early menopause, ovariectomy by age 50, avoiding dairy products, low exposure to sunlight, appear to be significantly associated with lower T-score values (Chi^2^ = 9.702, *p* < 0.01). Physical inactivity and scarce sun exposure also show to have a certain role, although not statistically significant (Chi^2^ = 3.423, *p* = 0.064 and Chi^2^ = 3.650, *p* = 0.056 respectively).

**Table 4:** Logistic regression evaluating discretized T-score effect versus nominal risk factors: likelihood ratio test

Factor	Chi-square	Significance
Female sex	255.97	*p.* < 0.01
Family history of osteoporosis	3.11	*p.* < 0.01
Previous fracture	1.51	*p.* < 0.01
Underweight	20.04	*p.* < 0.01
Steroids	7.21	*p.* < 0.05
Rheumatoid Arthritis	2.86	ns
Nephrolithiasis*	3.00	ns
Thyroid*	3.85	ns
Early menopause	47.36	*p.* < 0.01
Amenorrhea > 12 months	0.15	ns
Ovariectomy by age 50	44.61	*p.* < 0.01
Alcohol consumption	0.11	ns
Smoking habit	6.18	*p.* < 0.05
Exercise < 30 min/day	0.17	ns
Avoiding dairy products	10.50	*p.* < 0.01
Exposure to sunlight	8.85	*p.* < 0.05
Crohn disease*	0.74	ns
Celiac disease*	0.31	ns
Rectocolitis ulcerosa*	0.40	ns
Secondary osteoporosis*	15.20	*p.* < 0.01

*As calculated in BT12 group (subset of *n* = 3526 subjects)

## Discussion

Osteoporosis-related fractures are among the main causes of adult disability in developed countries. However, bone fragility is still poorly recognized and treated, since it may remain asymptomatic for years. For these reasons, campaigns to assess bone status should be carried out in the general population in order to identify “at-risk” individuals. With this purpose, our screening program was conducted to identify subjects at risk for fragility fractures in order to plan appropriate further testing and treatment. The BoneTour campaign targeted all the subjects who spontaneously underwent assessment of fracture risk by means of ultrasound densitometry and clinical questionnaire. It involved as much as *n* =7305 subjects throughout 21 Italian cities. As regards bone status, this campaign is comparable to ESOPO study, by which the clinical usefulness of quantitative ultrasound was assessed for the first time in Italy (2000) to define the prevalence of osteoporosis and osteopenia and their association with fractures [15]. In the BoneTour sample, 18.1 % of the subjects (20.2 % of total women and 7.4 % of total men) were osteoporotic according to ultrasound T-score values, while 42.1 % (44.1 % of total women and 32.4 % of total men) were osteopenic.

The majority of the sample (83.7 %) was composed by women, and this reflects the common awareness that female gender commonly perceives an higher risk of experiencing osteoporosis and fragility fractures, either because of a smaller muscle and bone mass compared to male, either because of the sudden drop in estrogens that occurs with the onset of menopause. Indeed, women in their 50s were the mainly represented category.

As a direct evidence of this difference between genders, the average FRAX-A risk calculated for the subsample of men resulted lower than that of women, despite the habit of smoke and alcohol assumption resulted more incident among males. The older mean age of males compared to females was probably due to the fact that in the collective opinion osteoporosis is still seen as a threat only for women.

The average age of the subjects who took part in the screening was 58.3 years, while an earlier check of bone status would really be useful, in particular in the presence of a risk factor. The increased risk of fracture with age is only partly due to the reduction in BMD. Poor balance and muscle hypotonia have an obvious role in the elderly because they favor both sedentary lifestyle and risk of falls. In addition, with advancing age, vitamin D insufficiency/deficiency is becoming increasingly common and important for bone health [21]. We acknowledge that the incidence of falls or the muscular performance were not taken into account in the present analysis, and vitamin D status was only inferred by evaluation of sun exposure.

The BoneTour database confirmed the heavy impact that the parental history of osteoporosis has on skeleton health [22]. Interestingly, as much as 30,5 % of people under fifty years of age referred a family history of fracture, and this prevalence seems to decrease with aging.

Analogously, most of fractured subjects declared to have one or more notorious risk factor, such as alcohol excessive intake, use of steroids, RA, oophorectomy before 50. In turn, previous fractures were significantly correlated with a reduced QUS T-score, and consequently a higher risk of further fractures [23].

Underweight resulted associated with reduced bone stiffness. In this respect, different studies reported conflicting data on the influence of BMI on BMD. A Mexican study on postmenopausal women either with normal weight, overweight, or different degrees of obesity revealed that a positive and significant association between BMI and BMD at lumbar spine, total hip and femoral neck measured by DXA [24]. However, as reported in recent literature, visceral adiposity has been shown to be an independent inverse determinant of bone density in overweight subjects. This association may be mediated by adipokines and a chronic inflammatory state [25]. Other authors examined the relationship of serum leptin concentration with age, body weight, BMI and BMD in mainland Chinese women, finding that age-related changes in serum leptin concentration depends on BMI, but is not a direct determinant of BMD [26]. Regarding underweight, a study on adolescent female athletes found that, although weight-bearing exercise is typically associated with an increase in BMD, amenorrheic athletes have lower BMD than eumenorrheic athletes and nonathletic controls, likely as a consequence of low energy availability and subsequent hypogonadism [27].

Notably, as much as 8.3 % of women aged 40–49 reported the history of amenorrhea lasted over 12 months.

The loss of bone mass associated with early menopause is the result of the catabolic effect of estrogen deficiency, which sums the linear age-related loss of bone mass [28]. The earlier the cessation of ovarian activity occurs, the longer the exposition of the skeleton to the risk of trauma, and the greater the loss of BMD in the years to come. About 79 % of surveyed women were in menopause. The average age of onset of menopause was found to be 49,3 years (SD 4,6). Almost 12 % of them had an early menopause (≤45 years), with brief or absent hormone replacement therapy (HRT). As much as 37.4 % of early postmenopausal women resulted osteopenic, and 22.8 % osteoporotic.

Tobacco smoking appeared to be the most widespread behavioral risk factor, as regarding 38 % of the sample.

Combined analysis of studies involving about 60,000 people in Canada, USA, Europe, Australia and Japan shows that the smoking habit increases the risk of hip fracture by 1.5 times [29]. Although the risks associated with smoking grow along with age, the effects of cigarette smoking begin to occur early. Swedish studies showed, in a group of young male smokers between 18 and 20 years, a reduction in BMD and thinning of cortical bone, the component that gives most of its strength. Thus, at a young age, smoke reduces the bone mass peak, increasing the risk of osteoporosis in later years [30]. As for alcohol assumption, the risk of fracture related to smoking is in part due to loss of bone stiffness, especially in women after menopause. Studies in the UK suggest that postmenopausal smokers have a much more rapid decline in BMD [31]. In our sample, the larger proportion of smokers regarded the age range 50–59 (42.2 %).

One of the main behavioral protective factors is physical activity. Besides maintaining bone strength, the main goal of exercise is to increase muscle mass and function, as well as to maintain good balance and strength, in order to prevent falls. In particular, resistance exercises become even more important with increasing age, and besides it is difficult to build bone mineral after adulthood, exercise has been shown to lead to modest increases in BMD (1–2 %) [32]. Moreover, the correction of sedentary lifestyle would even allow health gain as regards the prevention of diabetes, overweight and cardiovascular disease.

Over 19 % of the people interviewed admitted to perform less than 30 min of physical activity per day, including walking, gardening and housework. When related to T-score, exercise factor shows a *p*-value very close to statistical significance.

It is not surprising that daily exposure to sunlight, crucial for the synthesis of vitamin D, did not last more than 10 min for over 24 % of the people. The scarce exposure to sunlight may belong to a vicious cycle that characterizes various pathological conditions affecting the developed countries population. Indeed, sunlight induces specialized light-sensitive retinal ganglion cells to release glutamate in the suprachiasmatic nucleus, and targets depression-associated neurotransmitter systems (serotonin, noradrenalin, and dopamine) [33]. Psychological distress, so prevalent in our society, has been associated both with health-damaging behaviour, such as excessive assumption of alcohol, smoking, sedentary and unhealthy diet, both with inflammation, all of which could increase the risk of developing a variety of pathologies [34], such as metabolic syndrome, cerebro- and cardio-vascular diseases [35], as well as osteoporosis [36]. In turn, the abovementioned pathologies can lead people not to perform exercise, especially outdoors, further increasing the distress condition.

Patients with RA display an increased risk of osteoporosis and fragility fractures [36]. Indeed this disease affected 2.38 % of our sample and presented a strong correlation with the history of fragility fracture (*p* < 0.01), independently of steroid use.

Considerably, also each of the other causes of secondary osteoporosis [37–40], i.e., Crohn’s disease, ulcerative colitis, celiac disease, hypercalciuria and hyperthyroidism, resulted as statistically significant in conditioning both QUS T-score and FRAX-A and –B.

These conditions should therefore be included in the assessment of fracture risk by FRAX®, in analogy with what is reported in the English algorithm Q-fracture. This software reports, as binary variables, “Gastrointestinal conditions likely to result in malabsorption (that is Crohn’s disease, ulcerative colitis, celiac disease, steatorrhoea, blind loop syndrome)” and “Other endocrine conditions (thyrotoxicosis, primary or secondary hyperparathyroidism, Cushing’s syndrome)” [41].

In literature controversial data exist about the possible role of the number of pregnancies and breastfeeding duration in influencing the availability of calcium to the maternal bone [42]. In our sample, the number of pregnancies and the duration of lactation did not affect QUS T-score significantly, probably because the environmental conditions protect mothers from calcium deficiency.

In summary, the results of the BoneTour 2012 campaign confirmed the influence of all the variables considered by FRAX algorithm on bone status (female gender, age, family history of osteoporosis, previous fracture, underweight, alcohol abuse, secondary osteoporosis and RA). Further factors, such as sunlight exposure, hyperthyroidism, calcium nephrolithiasis, ovariectomy before age 50, physical inactivity, have shown a correlation with a high FRAX-score and reduced T-score.

The original version of FRAX™, based on five regional estimates of hip fracture risk undertaken up to 20 years previously, has been updated in 2013 on the basis of the new fracture incidence rates reported in the Italian national hospitalization database for the year 2008 [43]. The revision resulted close to the original version, except for a lower 10-year probability estimated in the younger age groups, and a higher one in the oldest [43].

To verify fracture risk evaluated by BoneTour campaign, a 10-year follow-up of the whole sample should be performed, assessing the incidence of osteoporotic fracture, as done by Lapi and colleagues [44], who followed up a selected group of patients (aged 50–85) of 900 Primary Care Physicians until the occurrence of a fragility fracture. This study revealed a positive association between advanced age, history of fracture, use of steroids, rheumatoid arthritis, underweight (BMI < 20) and gastrointestinal disease, thus according to our data, as well as depression, liver disease, chronic obstructive pulmonary disease, use of anticonvulsants [44].

In light of these data, the accuracy of FRAX™ could be improved by comparing in the long term the incidence of occurring fractures to the risk predicted by the calculator; epidemiological studies like BoneTour campaign, assessing bone status, history of fractures and clinical parameters known to influence fracture risk, should be used to increase the reliability of the algorithm.

## Conclusions

The prevalence rate of osteoporosis was approximately 18.7 %, while the rate of osteopenia was about 42.6 %. The 10-year-fracture-risk calculated by FRAX™ algorithm displayed variation across Italy. The results confirmed the importance of variables already present in FRAX™ (female gender, age, family or personal history of osteoporotic fracture, alcohol use, steroids, rheumatoid arthritis), but showed that secondary forms of osteoporosis (Crohn disease, ulcerative colitis, hyperthyroidism, calcium nephrolithiasis), poor sun exposure, and of oophorectomy before age 50 added to FRAX prediction. These conditions deserve to be taken into account by the physician since they may impact bone strength. A screening campaign, as well as being helpful for the people to assess their bone status, raise awareness and learn how to prevent osteoporosis, is useful for the health system, which can then modify the intervention at the territorial and hospital level depending on the presence of specific risk factors in that region.
